# The original sin of unawareness of Alzheimer's disease

**DOI:** 10.1055/s-0044-1800815

**Published:** 2024-12-20

**Authors:** Gilberto Levy

**Affiliations:** 1 Independent Researcher, Rio de Janeiro RJ, Brazil.

The long path toward recognition of Alzheimer's disease (AD) as the main cause of dementia was driven early on by some notable neuropathologists. It may also be said that it was subjected to a major detour by some illustrious precursors of modern psychiatry. When Alois Alzheimer published in 1907 his seminal clinico-pathological case report and first described neurofibrillary tangles, he worked at the University of Munich under Emil Kraepelin. In the same year, although senile plaques had been previously known, they were extensively described and considered a specific finding of “senile dementia” by Oskar Fischer, working at the German University of Prague under Arnold Pick. 1

The concept of “senile dementia” originated in the 18 ^th^ century. In 1776, the Scottish physician William Cullen proposed a classification of diseases into four classes, one of which was entitled neuroses (in the sense of nervous diseases) and included “ *amentia senilis* ”, defined as a “decay of perception and memory, in old age”. However, the more detailed definition was given in 1838 by Jean-Étienne Esquirol, a favorite student of Philippe Pinel regarded as having established the foundation of the modern classification of mental diseases. 2 3 In the second tome of the treatise “ *Maladies Mentales: Considérées sous les Rapports Médical, Hygiénique et Médico-Légal* ” Esquirol 4 wrote:

“Senile dementia is the result of the progress of age. Man, imperceptibly pushed towards old age, loses his sensitivity with respect to the free exercise of the faculties of understanding, before arriving at the last degree of decrepitude. Senile dementia establishes itself slowly. It begins with the weakening of memory, particularly of the memory of recent impressions. Sensations are weak; attention, at first tiring, becomes impossible; the will is uncertain and without impulse; the movements are slow and impossible”.

Although Alzheimer had doubts about whether the clinico-pathological findings described by him in a patient with disease onset at age 51 years were distinct enough to be considered separately from senile dementia, Kraepelin considered the findings from that case, and additional ones with age of onset under 65 years described by two Italian neuropathologists associated with Alzheimer (Francesco Bonfiglio and Gaetano Perusini), as a form of “presenile dementia”, and proposed that the condition be named after Alzheimer. Thus, in 1910, in the second volume of the eighth edition of his influential textbook, Kraepelin introduced “Alzheimer's disease” into the medical literature as a distinct entity from senile dementia, even if he also expressed his own doubts about that. 1 2

All of which is to point out that only a small minority of the cases we now call AD (early-onset AD) corresponds to the initial meaning of the eponymic term introduced by Kraepelin. The large majority of AD cases (late-onset AD) were regarded as part of senility or an inevitable consequence of aging. In this sense, one may consider that the unawareness or lack of recognition of AD among healthcare professionals is an “original sin”.

In this issue of *Arquivos de Neuro-Psiquiatria* , Malaga et al. 5 reports on the knowledge of dementia and AD among healthcare professionals in Peru. The authors analyzed 748 surveys of a diverse group of healthcare professionals (the vast majority comprising ∼40% of medical doctors and 50% of nurses or nursing technicians), working both in primary care centers and tertiary care hospitals. About 75% of the respondents worked in the country capital, Lima, and 25% in smaller cities. The main findings of the study were characterized by the authors as surprising or even “astounding”. Asked to name the types of dementia they were familiar with, only ∼25% of respondents (28,6% of physicians) knew more than one type. When they were specifically asked if AD was a type of dementia, ∼20% said no (∼15% of physicians). Also worrisome was that almost 30% of respondents or physicians, when given a list of 13 risk factors for AD, could not recognize more than two.

Malaga et al. 5 sounded particularly disturbed by the finding that, while 20% of respondents didn't recognize AD as a cause of dementia when specifically prompted about it, more than half (51.4%) spontaneously listed “senile dementia” as a type of dementia. The authors argued that the concept of senile dementia is obsolete and that even if “dementia is significantly more common in old age, it is not an inevitable consequence of aging”. More generally, one can argue that there is such a thing as normal or healthy aging, meaning aging in the absence of an aging-related disease. 6 That, in this day and age, senile dementia is still largely elicited as a type of dementia among healthcare professionals, to the apparent detriment of the recognition of AD, speaks to the original sin of unawareness of AD, so much so that the authors seem justified in calling senile dementia “a pernicious idea”. 5

The analysis also showed a higher level of knowledge of dementia and AD in Lima *versus* smaller cities in Peru with a much lower gross domestic product, and the authors cite studies supporting the generalizability of their findings to other developing Latin American countries. Yet studies also indicate that the problem of unawareness of AD among primary care physicians similarly afflicts developed countries. For example, in a study conducted in the United States before the turn of the century, only 40% of generalists ( *versus* 97% of experts) recognized AD as the most common cause of severe memory loss in people over age 65 years. 7 In a more recent study conducted in France, Germany, Japan, United Kingdom, and the United States, physician suspicion of AD was a trigger for diagnosis in only 20% of cases; caregivers were the main drivers of the diagnosis. 8 Based on a 2022 review about the knowledge and attitudes of primary care physicians regarding AD, the author noted that “timely diagnosis and quality care of people with AD or other dementia is more an exception than a rule in many parts of the United States”, such that “dementia diagnosis commonly occurs in the middle to later stages of the disease”. 9

The unawareness or low level of knowledge of AD among healthcare professionals poses several obstacles. First, as pointed out by Malaga et al., 5 difficulties with the detection of dementia at the primary care level, especially where referral to specialty care is not an option, “constitutes an obstacle for early diagnosis, which limits the possibility to preserve and improve the quality of life of patients and caregivers”. Second, the lack of knowledge about risk factors for AD constitutes an obstacle for primary prevention, “via public healthcare approaches focused on addressing key modifiable medical and lifestyle risk factors”. 5 Third, unawareness of AD among primary care physicians represents an obstacle for research studies recruitment in areas with no access to specialists (e.g., rural communities), which may affect study generalizability. 10 Finally, if an unequivocally effective disease-modifying treatment becomes available, which is not quite the case yet, a low level of knowledge of AD among healthcare professionals will represent an obstacle for early treatment.

In that hopeful scenario of an effective disease-modifying treatment, given that early initiation may be crucial for the effectiveness of a disease-modifying agent, the original sin of unawareness of AD will turn out to be wholly unforgivable.
